# Addition of a new *Quedius* Steph. (Coleoptera, Staphylinidae) species to the biodiversity of Albertan mixedwood forest, Canada

**DOI:** 10.3897/zookeys.668.12320

**Published:** 2017-04-12

**Authors:** Jenna M. Jacobs, J. A. Colin Bergeron

**Affiliations:** 1 Département des Sciences Biologiques, Université du Québec à Montréal, Pavillon des sciences biologiques (SB), 141 Avenue du Président-Kennedy, Montréal, Québec, Canada, H2X 1Y4; 2 Department of Renewable Resources, University of Alberta, 442 Earth Sciences Building, Edmonton, Alberta, Canada, T6G 2E3

**Keywords:** *Quedius spencei*, *Raphirus*, EMEND

## Abstract

Quedius (Raphirus) spencei Jacobs and Bergeron, new species, (Coleoptera: Staphylinidae), is described based on specimens from two localities (type locality: 35 km. E Dixonville, Alberta, Canada) in the Boreal Forest. Male genitalia are illustrated, compared with congeners (*Q. rusticus* Smetana and *Q. simulator* Smetana) in the *Aenescens* species group, and included in a slightly modified key to the species of *Quedius*.

## Introduction

The genus *Quedius* Stephens currently contains 92 species in America north of Mexico (Newton et al. 2001; Smetana and Webster 2011). Taxonomy of this genus has been revised three times by Horn (1871), Casey (1915) and Smetana (1971a, 1971b, 1973, 1976, 1978, 1981). The Alberta fauna of the genus *Quedius* is particularly diverse with 29 species (Bousquet et al. 2013) and has been included in many biodiversity studies in boreal forest (Bergeron et al. 2013; Buddle et al. 2006; Gandhi et al. 2004; Gandhi et al. 2001; Hammond et al. 2001, 2004; Jacobs et al. 2007a, 2007b; Pohl et al. 2008; Pohl et al. 2007). J. Jacobs detected a specimen of an undescribed species while identifying flight intercept trap samples from a central Albertan study on saproxylic beetles (Cobb et al. 2011). Further specimens (fifteen) of this same species were found from pitfall trap samples collected by C. Bergeron from a northwestern Albertan forest biodiversity study (Bergeron et al. 2011). In an effort to assess the impact of alternative forest management practices on the boreal mixedwood forest ecosystem, the Ecosystem Management Emulating Natural Disturbance (EMEND) research site is subject to long term intensive arthropod sampling. Therefore, this species has the potential to contribute important information about ecological processes and modern forest management techniques. Furthermore, the fact that this species was collected from two sites separated by c. 260 km suggests that it may be widely distributed at least in the boreal mixedwood forest of Alberta. Combined with the increasing popularity of ground dwelling and saproxylic beetles in ecological impact assessment (Langor and Spence 2006), and the widespread use of pitfall and flight intercept traps in such studies, it is very likely that this species will be collected again. For these reasons, as well as taxonomical interest, we describe the new species in this paper.

### Classification

In North America, the genus *Quedius* is divided into 6 subgenera. *Quedius spencei* is included in the subgenus Raphirus Stephens with 23 other species characterized by large eyes and a usually broad and narrowly bilobed labrum (Smetana 1971a). This new species belongs to the *Aenescens* group which is characterized by the two additional setiferous punctures between the anterior frontal punctures and the placement of the last puncture of sublateral row distinctly behind the level of the lateral puncture. The following description is based on the terms used by (Smetana 1971a) for the similar species *Quedius rusticus* Smetana and *Quedius simulator* Smetana.

## Methods

### Measurements and ratios

The width of the head was measured along the widest part including the eyes. The length was measured along the midline, from the base of the head to the apex of clypeus. These measurements were used to determine the width to length ratio (w:l) for the head. The length of the eyes (from anterior to posterior margin) and the length of the temples (from posterior margin of the eyes to the neck) were measured as viewed dorsally. These values were used to determine the temple to eye ratio (t:e). The width of the pronotum was measured along the widest segment separating the lateral margins of the pronotum and the length is measured along the midline from the anterior to the posterior margin. These measurements were used to calculate w:l ratio for the pronotum. The lateral length of the elytra was measured between the humeri and the posterior elytral angle. This measurement was used to define the elytra at sides to pronotum at midline ratio.

## Description

### Quedius (Raphirus) spencei

Taxon classificationAnimaliaColeopteraStaphylinidae

 Jacobs & Bergeron sp. n.

http://zoobank.org/7458C3CE-78E3-4AFE-9060-01FE507CCB7D

Figs 1 , 2A

#### Description.

**Figure 1.**
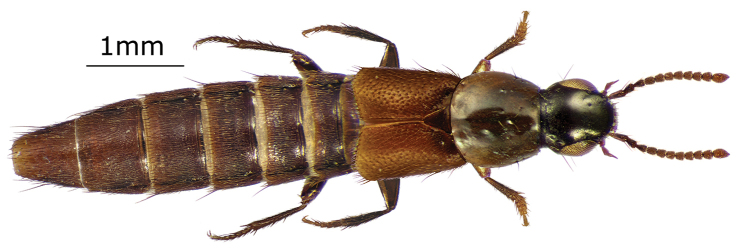
Dorsal habitus of *Quedius spencei*, sp. n.

Habitus as in Fig. 1. Piceous to piceous black, elytra and abdominal tergites of some specimens brownish. Palpi, antennae and legs piceous to brownish with tibia distinctly darker than rest of leg. Head, pronotum and elytra with bronze luster. Head rounded, slightly transverse (1.07–1.09 w:l). Eyes large, considerably longer than the length of the temples in dorsal aspect (0.31–0.32 t:e). Two additional setiferous punctures between anterior frontal punctures, posterior frontal puncture situated somewhat closer to posterior margin of eye than posterior margin of head (similar to *Q. rusticus*). Surface of head with very fine and dense microsculpture consisting of transverse lines. Antennae with first 3 segments darker and elongate (longer than wide), third segment slightly shorter than second, segments 4-11 densely pubescent. Segments 4 and 5 are slightly longer than wide, sixth barely longer than wide, and segments 7–10 quadrate to slightly transverse. Pronotum as long as wide (1.00, w:l), broadly arcuate at base and moderately narrowed in front. Chaetotaxy of pronotum similar to other species in the *Aenescens* group with three punctures in each dorsal row, sublateral rows with last puncture situated distinctly behind level of large lateral puncture; microsculpture similar to head. Scutellum impunctate. Elytra at sides only barely longer than pronotum at midline (1.07–1.10). Punctation and pubescence of elytra fine and moderately dense (as in *Q. rusticus*), interspaces smooth without distinct microsculpture. Punctation of abdominal tergites finer than punctuation of elytra and usually a little denser on bases of first three or four visible abdominal tergites. Pubescence brownish with a single large piceous bristle originating from the lateral apex of first four visible abdominal tergites, usually with a second bristle on the second to fourth visible tergites (as in *Q. rusticus* and *Q. simulator*).

#### Male.

**Figure 2.**
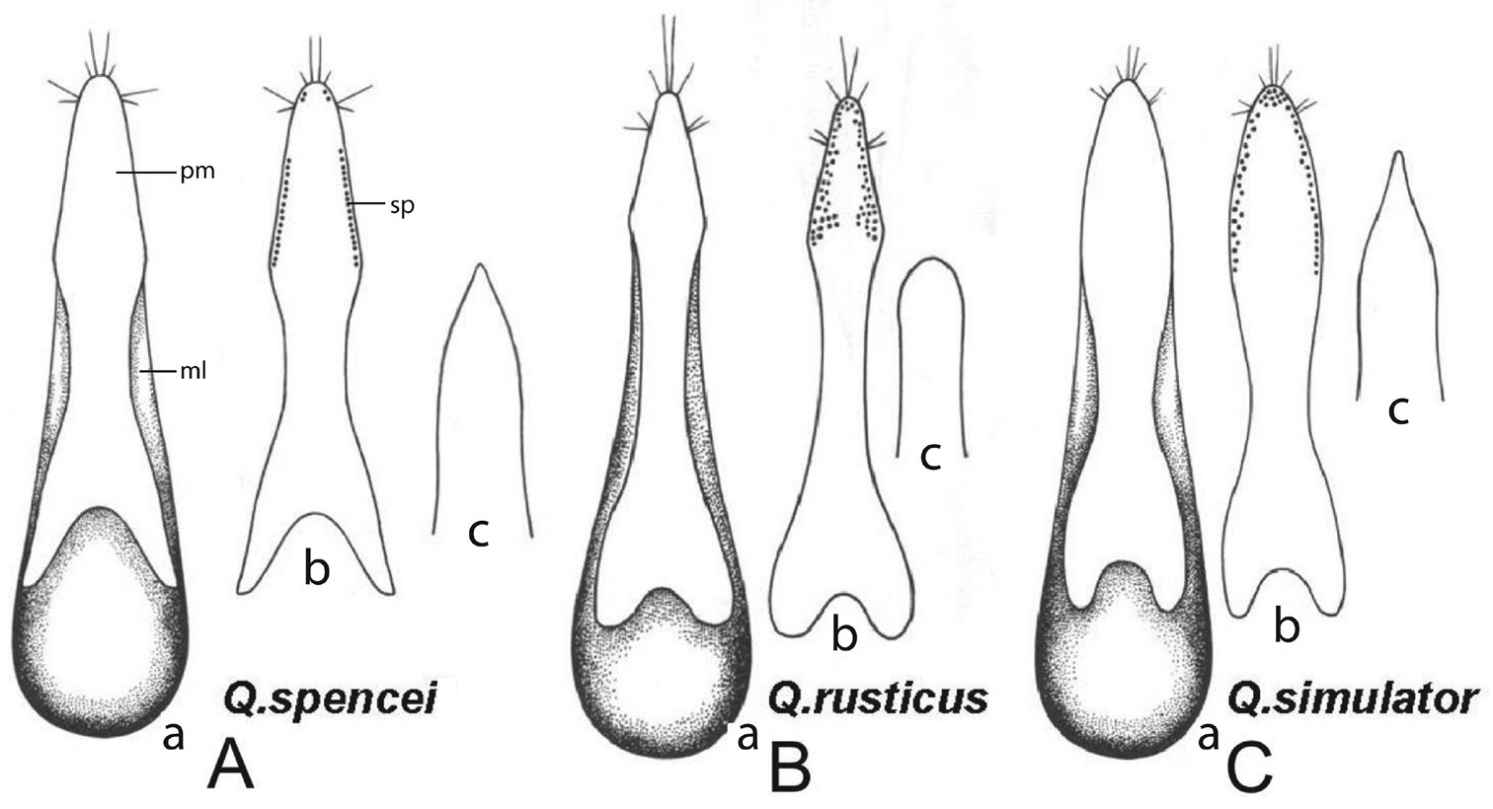
Male genitalia of **A** *Q. spencei* **B** *Q. rusticus*, and **C** *Q. simulator* **a** aedeagus, ventral aspect, ml: median lobe, pm: paramere **b** paramere, dorsal aspect, sp: sensory peg setae c) apex of median lobe, ventral aspect. Illustrations of *Q. rusticus* and *simulator* from Smetana (1971a).

Sixth visible sternite with a moderately shallow, obtuse triangular emargination in the middle of apical margin, with a slightly impressed, smooth, narrow triangular area anteriad of the emargination, less than twice the depth of the emargination. Aedeagus with paramere extended to the tip of narrow, sharp median lobe. Paramere slightly narrowed posteriad of base, expanded to maximum width one-third from apex, at which point margins obtusely angle toward narrowed apex (Fig. 2A; pm, a, b). Paramere with several short and two long apical bristles, with two additional long bristles on lateral margins near apex. Sensory peg setae on dorsal surface of paramere arranged in a single row on lateral margins, terminated distinctly anteriad of apex, with one to three additional peg setae on each side of apex (Fig. 2A; b, sp).

Length: 5.6–5.8mm.

#### Type material.

Type material is deposited in the Strickland Entomological Museum at the University of Alberta (UASM) and at the Canadian National Collection of Insects, Arachnids and Nematodes in Ottawa (CNC). See supplementary table for more information on each specimen.

*Holotype* (♂) // CAN:AB: EMEND, 56°46'13"N; 118°22'28"W, Coll: C. Bergeron 2003 // HOLOTYPE, *Quedius spencei* Jacobs & Bergeron, // CB1802 // UASM# 212610. The holotype is pointed with genitalia stored in plastic vial. The right foreleg is missing tibia and tarsus and the right hind leg is missing the three last tarsal segments. Left maxillary palp is broken.

Paratypes: 7 ♂♂, same labels as holotype but CB0954 // UASM# 212609; CB1555 // UASM# 212608; CB1555 // UASM# 212606; CB0956 // UASM# 212607; CB0955 // UASM# 212611; CB2034 // CNC# 615416; CB2036 // CNC# 615417; and 3 ♂♂ CAN:AB: Slave Lake, 55°17'52"N; 115°05'29"W, Coll. T.Cobb 2003 // PARATYPE, *Quedius spencei*, Jacobs & Bergeron // tpc02841 // CNC# 615418; tpc02320 // CNC# 615419; tpc3220 // CNC# 615420.

#### Type locality.

EMEND research site, 35 km east of Dixonville, Alberta, Canada

#### Geographical distribution.

Known specimens were collected from central Alberta, Slave Lake (N55°17.86', W115°05.49') and north-western Alberta, near Dixonville (N56°46,22', W118°22.47'). Probably more widely distributed.

#### Collection notes.

The specimens from Dixonville were collected in pitfall traps operating from the second week of May until the last week of June 2003 in old-growth spruce-fir forest. The Slave Lake specimens were collected from a flight intercept trap in May 2003 in a former conifer forest two years following harvesting; regenerating with aspen trees. *Quedius spencei* seems to live in conifer forest.

#### Comparison and diagnostic features.

*Quedius spencei* is similar in general habitus to *Quedius rusticus* Smetana, but shares characteristics of the male genitalia with *Quedius simulator* Smetana: shape of the apex of the median lobe acute (Fig. 2A, c), and paramere of the male genitalia does not exceed the apex of the median lobe. However, *Q. spencei* differs from *Q. simulator* in other characters of the aedeagus: paramere (Fig. 2A; pm, a, b) with margins of apical half obtusely angulate, reaching maximum width one-third from apex; base of paramere deeply emarginate (Fig. 2A, b); and sensory peg setae (Fig. 2A, b, sp) on each lateral margin in a single row. Additionally, similar to *Q. rusticus* this species is lighter in coloration than *Q. simulator*, with elytra usually brownish, and apical margins of tergites and apex of abdomen paler. Also, similar to *Q. rusticus*, the posterior frontal puncture of head is removed from the hind margin of eyes.

#### Etymology.

The specific epithet is an eponym, a singular Latin noun, genitive case, based on the surname of our mentor and friend, John R. Spence, who has dedicated his career to the study of arthropod biodiversity, particularly ecological and taxonomic aspects of gerrid bugs and carabid beetles, and to community aspect of forest invertebrates. He has mentored many students, putting emphasis on species level identifications, and has greatly influenced the field of entomology in Canada.

### Key to selected species of *Quedius* (modified from Smetana 1971a)

**Table:** 

21(22)	Paramere with the apex considerably exceeding the apex of median lobe, which is broadly arcuate (Fig. 2B, c)	***Q. rusticus* Smetana**
22(21)	Paramere with the apex about even with the apex of median lobe, which is more or less acute (figs 136, 137, Smetana 1971a).
22a(22b)	Paramere with margins of apical half obtusely angulate (Fig. 2A, a,b). Coloration usually lighter, punctation of abdomen moderately dense. Length 5–6mm	***Q. spencei* sp. n.**
22b(22a)	Paramere with margins of apical half broadly rounded (Fig. 2C, a,b). Coloration usually darker, punctation of abdomen usually very dense.
23(24)	Paramere with the apical part long and spatulate, the narrow and more or less parallel-sided middle part short (Fig. 2C, a,b). Head and pronotum usually with darker metallic luster; punctation of abdomen usually finer and more dense. Length 5–7mm	***Q. simulator* Smetana**
24(23)	Paramere with the apical part short, lancet-like, the narrow and more less parallel-sided middle part long (fig. 137, Smetana 1971a). Head and pronotum usually with lighter metallic luster; punctation of abdomen usually less fine and dense. Length 5–7mm	***Q. aenescens* Mäklin**

## Supplementary Material

XML Treatment for Quedius (Raphirus) spencei

## Supplementary materials

Supplementary material 1 Locality table for the holotype and paratype specimens of *Quedius spencei* Jacobs & Bergeron, sp. n. Data type: occurence This dataset is made available under the Open Database License (http://opendatacommons.org/licenses/odbl/1.0/). The Open Database License (ODbL) is a license agreement intended to allow users to freely share, modify, and use this Dataset while maintaining this same freedom for others, provided that the original source and author(s) are credited.Jenna M. Jacobs, J. A. Colin Bergeron

## References

[B1] Bergeron JAC Spence JR Volney WJA (2011) Landscape patterns of species-level association between ground-beetles and overstory trees in boreal forests of western Canada (Coleoptera, Carabidae). ZooKeys: 577–600. https://doi.org/10.3897/zookeys.147.2098 22371676 10.3897/zookeys.147.2098 PMC3286240

[B2] Bergeron JAC Spence JR Volney WJA Pinzon J Hartley DJ (2013) Effect of habitat type and pitfall trap installation on captures of epigaeic arthropod assemblages in the boreal forest. The Canadian Entomologist 145: 547–565. https://doi.org/10.4039/tce.2013.38

[B3] Bousquet Y Bouchard P Davies A Sikes D (2013) Checklist of beetles (Coleoptera) of Canada and Alaska. Second edition. Pensoft Publishers, Sofia, Bulgaria, 402 pp. 10.3897/zookeys.360.4742 PMC3867111 24363590

[B4] Buddle CM Langor DW Pohl GR Spence JR (2006) Arthropod responses to harvesting and wildfire: Implications for emulation of natural disturbance in forest management. Biological Conservation 128: 346–357. https://doi.org/10.1016/j.biocon.2005.10.002

[B5] Casey TH (1915) Studies in some staphylinid genera of North America. In: Casey TH (Ed.) Memoirs on the Coleoptera, 6. The New Era Printing Co., Lancaster, PA, 395–450.

[B6] Cobb TP Morissette JL Jacobs JM Koivula MJ Spence JR Langor DW (2011) Effects of Postfire Salvage Logging on Deadwood-Associated Beetles. Conservation Biology 25: 94–104. https://doi.org/10.1111/j.1523-1739.2010.01566.x 20735453 10.1111/j.1523-1739.2010.01566.x

[B7] Gandhi KJ Spence JR Langor DW Morgantini LE Cryer KJ (2004) Harvest retention patches are insufficient as stand analogues of fire residuals for litter-dwelling beetles in northern coniferous forests. Canadian Journal of Forest Research 34: 1319–1331. doi:https://doi.org/10.1139/x04-018

[B8] Gandhi KJK Spence JR Langor DW Morgantini LE (2001) Fire residuals as habitat reserves for epigaeic beetles (Coleoptera: Carabidae and Staphylinidae). Biological Conservation 102: 131–141. https://doi.org/10.1016/s0006-3207(01)00099-4

[B9] Hammond HEJ Langor DW Spence JR (2001) Early colonization of Populus wood by saproxylic beetles (Coleoptera). Canadian Journal of Forest Research 31: 1175–1183. https://doi.org/10.1139/x01-057

[B10] Hammond HEJ Langor DW Spence JR (2004) Saproxylic beetles (Coleoptera) using Populus in boreal aspen stands of western Canada: spatiotemporal variation and conservation of assemblages. Canadian Journal of Forest Research 34: 1–19. https://doi.org/10.1139/x03-192

[B11] Horn GH (1871) Descriptions of New Coleoptera of the United States, with Notes on Known Species. Transactions of the American Entomological Society (1867–1877) 3: 325–344. https://doi.org/10.2307/25076255

[B12] Jacobs JM Spence JR Langor DW (2007a) Influence of boreal forest succession and dead wood qualities on saproxylic beetles. Agricultural and Forest Entomology 9: 3–16. https://doi.org/10.1111/j.1461-9563.2006.00310.x

[B13] Jacobs JM Spence JR Langor DW (2007b) Variable retention harvest of white spruce stands and saproxylic beetle assemblages. Canadian Journal of Forest Research 37: 1631–1642. https://doi.org/10.1139/X07-020

[B14] Langor DW Spence JR (2006) Arthropods as ecological indicators of sustainability in Canadian forests. The Forestry Chronicle 82: 344–350. https://doi.org/10.5558/tfc82344-3

[B15] Newton AF Thayer MK Ashe JS Chandler DS (2001) Staphylinidae, Latreille, 1802. In: Arnett RH Thomas MC (Eds) American beetles, volume 1, Archostemata, Myxophaga, Adephaga, Polyphaga: Staphyliniforma. CRC Press, Boca Raton, 272–418.

[B16] Pohl G Langor D Klimaszewski J Work T Paquin P (2008) Rove beetles (Coleoptera: Staphylinidae) in northern Nearctic forests. The Canadian Entomologist 140: 415–436. https://doi.org/10.4039/n07-LS03

[B17] Pohl GR Langor DW Spence JR (2007) Rove beetles and ground beetles (Coleoptera: Staphylinidae, Carabidae) as indicators of harvest and regeneration practices in western Canadian foothills forests. Biological Conservation 137: 294–307. https://doi.org/10.1016/j.biocon.2007.02.011

[B18] Smetana A (1971a) Revision of the tribe Quediini of America north of Mexico (Coleoptera: Staphylinidae). Memoirs of the Entomological Society of Canada 79: 1–303. https://doi.org/10.4039/entm10379fv

[B19] Smetana A (1971b) Revision of the tribe Quediini of America north of Mexico (Coleoptera: Staphylinidae), Supplementum. The Canadian Entomologist 103: 1833–1848. https://doi.org/10.4039/Ent1031833-12

[B20] Smetana A (1973) Revision of the tribe Quediini of America north of Mexico (Coleoptera: Staphylinidae), Supplementum 2. The Canadian Entomologist 105: 1421–1434. https://doi.org/10.4039/Ent1051421-11

[B21] Smetana A (1976) Revision of the tribe Quediini of America north of Mexico (Coleoptera: Staphylinidae), Supplementum 3. The Canadian Entomologist 108: 169–184. https://doi.org/10.4039/Ent108169-2

[B22] Smetana A (1978) Revision of the tribe Quediini of America north of Mexico (Coleoptera: Staphylinidae), Supplementum 4. The Canadian Entomologist 110: 815–840. https://doi.org/10.4039/Ent110815-8

[B23] Smetana A (1981) Revision of the tribe Quediini of America north of Mexico (Coleoptera: Staphylinidae), Supplementum 5. The Canadian Entomologist 113: 631–644. https://doi.org/10.4039/Ent113631-7

[B24] Smetana A Webster RP (2011) A new species of the genus Quedius Stephens, 1829, subgenus Microsaurus Dejean, 1833, from northeastern North America (Coleoptera, Staphylinidae, Staphylinini, Quediina). ZooKeys: 39–47. https://doi.org/10.3897/zookeys.126.1647 10.3897/zookeys.126.1647 PMC3175133 21998540

